# Supplementary material for: The causal relationship between the human gut microbiota and pyogenic arthritis: a Mendelian randomization study
Source: Front Cell Infect Microbiol. 2024 Nov 26;14:1452480. doi: 10.3389/fcimb.2024.1452480 (PMC11629706; doi:10.3389/fcimb.2024.1452480)
Supplement: Supplementary file 1 [file DataSheet1.zip › mendelian test/ebi-a-GCST90027488.csv_forest.pdf]

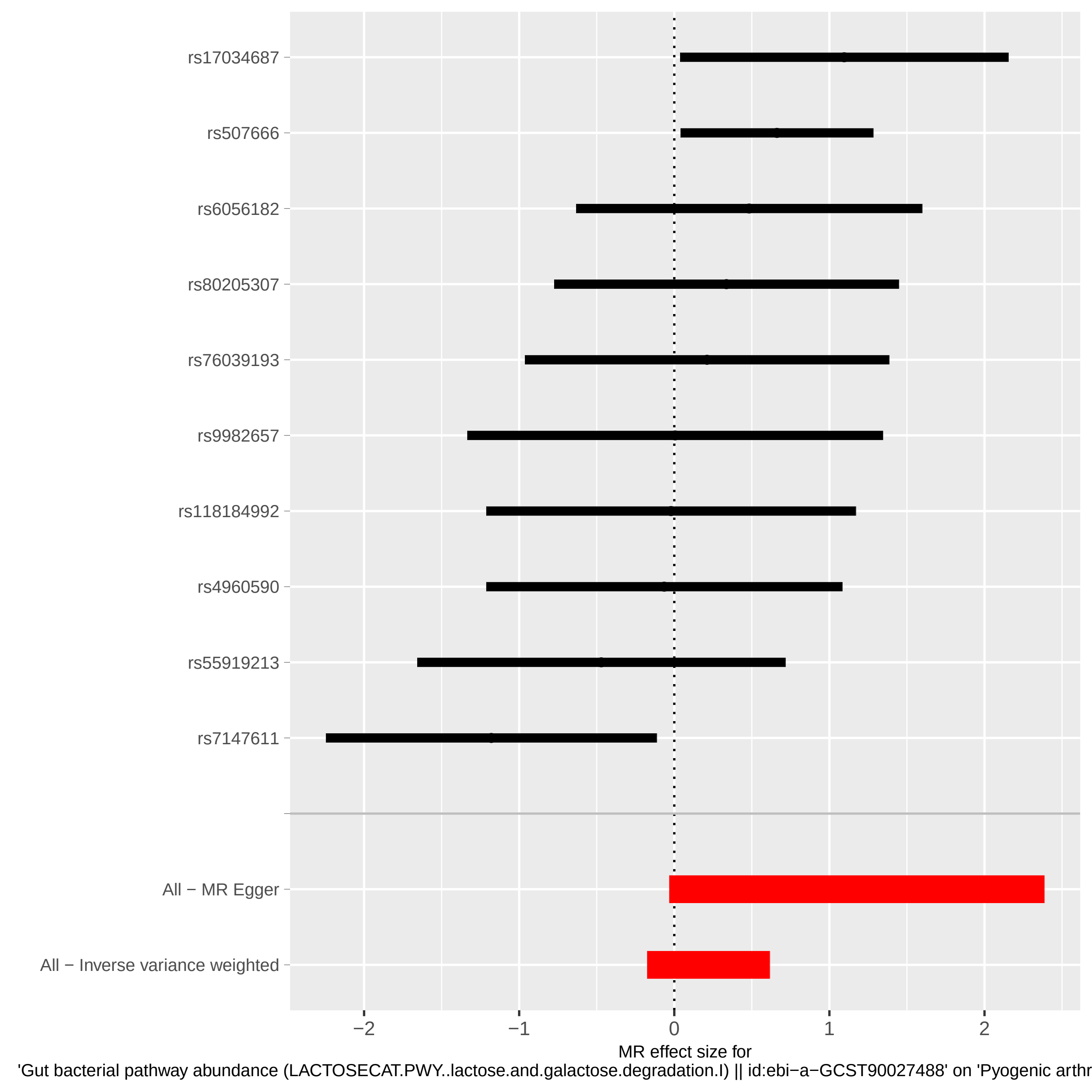
rs17034687
rs507666
rs6056182
rs80205307
rs76039193
rs9982657
rs118184992
rs4960590
rs55919213
rs7147611
All − MR Egger
All − Inverse variance weighted
−2
−1
0
1
2
MR effect size for
'Gut bacterial pathway abundance (LACTOSECAT.PWY..lactose.and.galactose.degradation.I) || id:ebi−a−GCST90027488' on 'Pyogenic arthr
